# Volvulus du cæcum: une cause rare d’occlusion intestinale: à propos de deux cas

**DOI:** 10.11604/pamj.2017.28.162.12237

**Published:** 2017-10-19

**Authors:** Khalid Mazine, Hicham Elbouhaddouti, Imane Toughrai, Ouadie Mouaqit, Elbachir Benjelloun, Abdelmalek Ousadden, Khalid Ait Taleb

**Affiliations:** 1 Service de Chirurgie Viscérale A(C3), CHU Hassan II, Fès, Maroc; 2 Service de Chirurgie Viscérale B(E3), CHU Hassan II, Fès, Maroc

**Keywords:** Occlusion intestinale, volvulus, cæcum, résection iléo-cæcale, Intestinal occlusion, volvulus, cecum, ileocecal resection

## Abstract

Le Cæcum est, en fréquence, la deuxième partie du colon concernée par le volvulus après le sigmoïde et avant l'angle gauche et le côlon transverse. Cette affection survient sur des cæcums anormalement mobiles. Le mécanisme du volvulus est la torsion ou la bascule. Le tableau clinique est celui d'une occlusion intestinale aiguë par strangulation. L'abdomen sans préparation (ASP) et la TDM abdominale sont les examens radiologique de premier choix pour le diagnostic. Le traitement consiste en une chirurgie en urgence avec résection du cæcum et de l'iléon terminal. Nous rapportons deux cas de volvulus du cæcum admis aux urgences dans un tableau d'occlusion intestinale aiguë, le diagnostic était confirmé chez les deux patients par un scanner abdomino-pelvien et le traitement consistait en une résection iléo-colique avec rétablissement immédiate de continuité, les suites post opératoires étaient simples.

## Abstract (en)

The cecum is the second part of the colon that is most commonly affected by the volvulus after sigmoid colon and before left corner and the transverse colon. This condition occurs in patients with abnormally mobile cecum. Volvulus is characterized by torsion or tilt. Clinically, it appears as bowel obstruction due to acute strangulation. Abdominal x-ray without treatment and abdominal CT scan are the radiological procedures of choice in the diagnosis of volvulus of the cecum. Treatment is based on emergency surgical excision of the cecum and of the terminal ileum. We report two cases of patients with volvulus of the cecum admitted to the emergency department with acute intestinal obstruction. In both patients, the diagnosis was confirmed by abdomino-pelvic CT scan and the treatment was based on ileocolic resection with immediate restoration of the intestinal continuity. The postoperative course was uneventful.

## Introduction

La première description de volvulus du cæcum a été rapportée par Rokitanski en 1837 [1]. Il s'agit d'une torsion de la partie initiale du côlon droit et de la partie terminale de l'iléon autour du pédicule vasculaire colique inférieur droit. Il serait responsable de 1% des occlusions intestinales [2]. Malgré de nombreuses publications la symptomatologie et la prise en charge de cette pathologie demeurent des sujets de controverse [2,3]. Nous rapportons l'observation de deux patients consécutifs ayant été pris en charge aux urgences pour un volvulus du cæcum.

## Patient et observation

**Figure 1:**
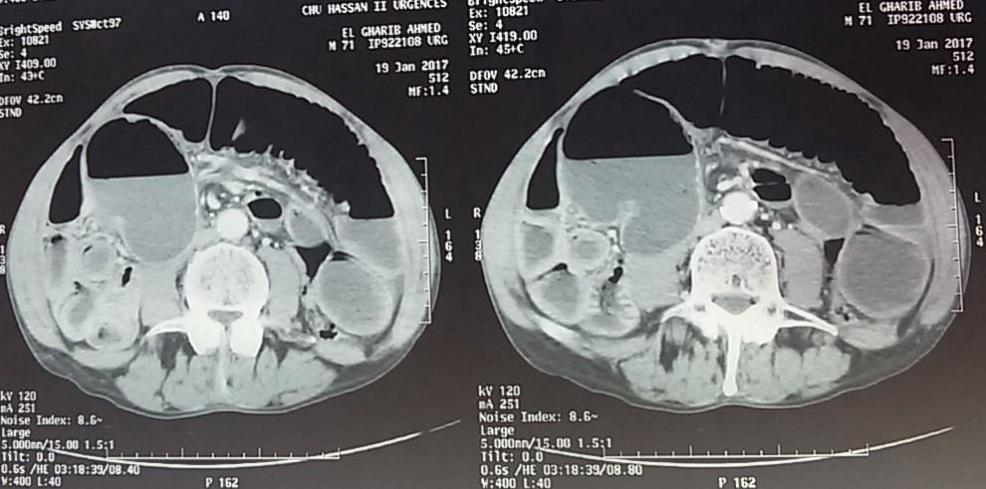
TDM abdominale premier patient

**Figure 2:**
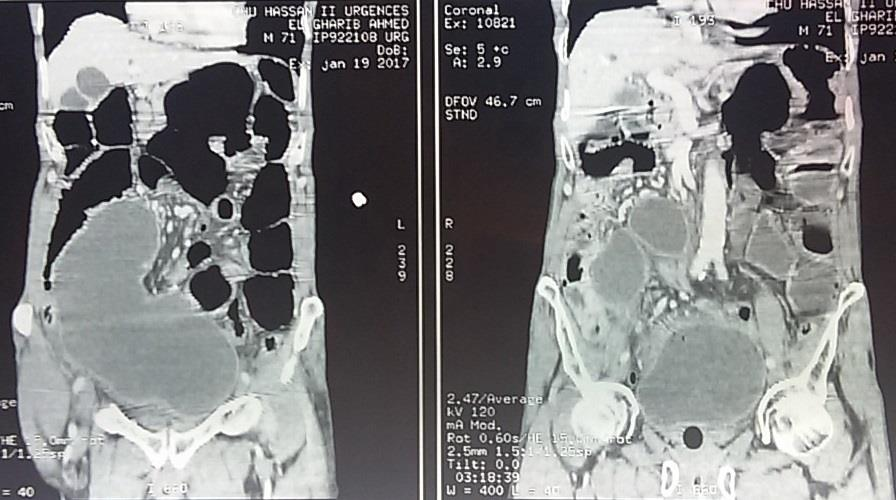
Reconstruction frontale TDM abdominale premier patient

**Figure 3:**
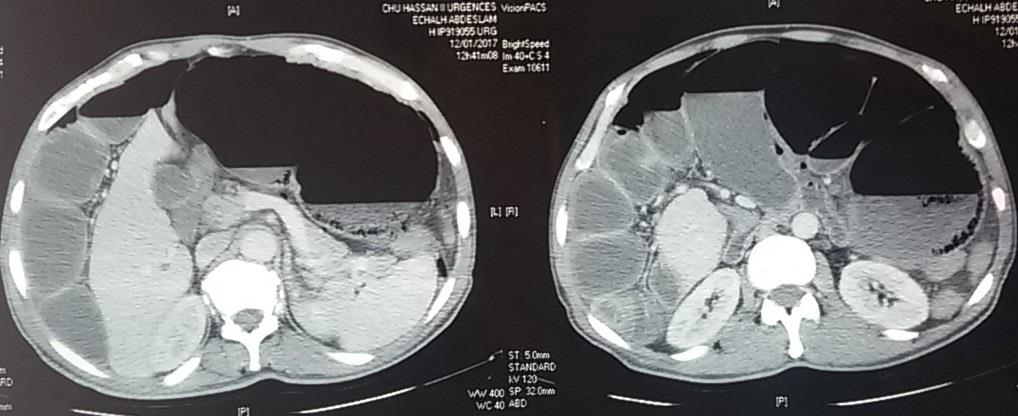
TDM abdominale deuxième patient

**Figure 4:**
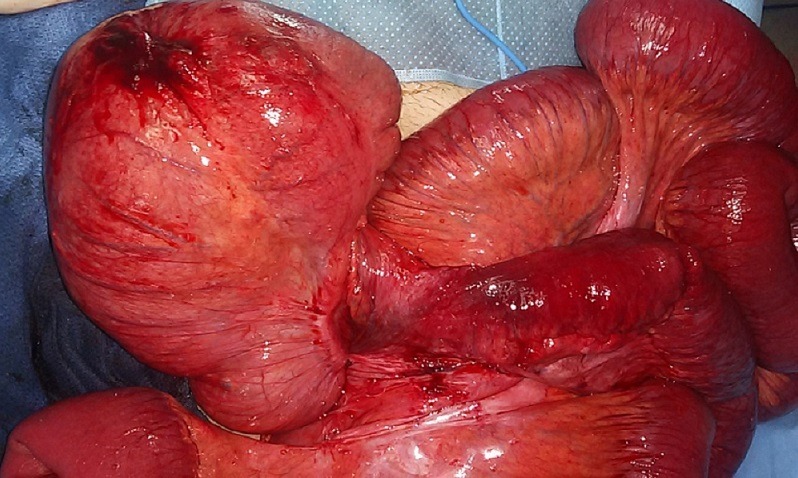
Image per opératoire premier patient

**Figure 5:**
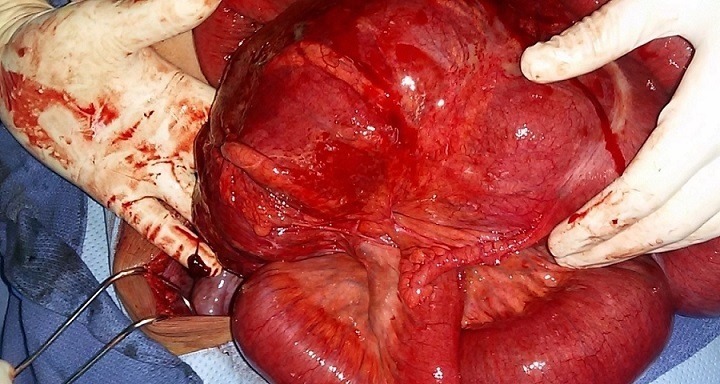
Image per opératoire deuxième patient

Deux patients âgés de 58 et 66 ans ont été admis aux urgences à quinze jours d'intervalle, pour un tableau d'occlusion avec arrêt des matières et des gaz, météorisme et douleur abdominale diffuse. L'interrogatoire a retrouvé un mode d'installation brutal, similaire pour les deux patients , avec une symptomatologie évoluant cinq jours avant la consultation pour le premier patient qui présente également des vomissements fécaloïdes et un intervalle de deux jours pour le deuxième patient qui ne présente pas de vomissements. L'examen notait un abdomen distendu, hypertympanique pour les deux patients avec une légère sensibilité abdominale diffuse, les orifices herniaires étaient libres et l'ampoule rectale vide. Le bilan biologique retrouvait une hyperleucocytose à prédominance PNN à 15 000 éléments/mm^3^ et 18 000 éléments /mm^3^ la CRP était élevé pour les deux patients à 150 et 220, la fonction rénale normal pour un patient et légèrement altérée pour l'autre. Les deux patients ont bénéficié d'un abdomen sans préparation en position debout objectivant des niveaux hydro-aérique de type grêlique. Ils ont ensuite bénéficié d'un scanner abdomino-pélvien injecté, il a objectivé une importante distension grêlique en amont d'un volvulus iléo-cæcal, un des patients avait un pneumo-péritoine avecun défaut de rehaussement au niveau de la paroi cæcale et un épanchement intra-péritonéal de moyenne abondance (Figure 1, Figure 2, Figure 3). Les deux patients ont été opérés aux urgences,abordé par laparotomie,un patient a bénéficié d'une résection iléo-cæcale avec anastomose iléo-colique término-latérale manuelle. L'autre a eu une hémicolectomie droite avec anastomose iléo-transverse término-laterale .Les suites post opératoires étaient simples (Figure 4, Figure 5).

## Discussion

Le volvulus du cæcum est une torsion du côlon droit autour de son axe mésen-térique qui n'est possible que si le côlon proximal est mobile. La mobilité excessive du cæcum est due à une rotation embryologique incomplète de l'intestin ou à un défaut d'accolement du côlon ascendant au péritoine pariétal postérieur [4,5]. Deux grands types de volvulus sont décrits: soit par une rotation du côlon autour de son axe, le cæcum restant alors dans le cadran abdominal inférieur droit, soit par une bascule du cæcum associée à une rotation du côlon qui se place alors dans le cadran supérieur gauche de l'abdomen [2,6,7]. Le diagnostic de volvulus du cæcum est difficile car les signes cliniques ne sont pas spécifiques et que l'intensité de la douleur est extrêmement variable [5]. Il se manifeste généralement par une occlusion digestive plus ou moins aiguë. Le cliché de l'abdomen sans préparation peut être utile pour le diagnostic mais sa sensibilité est généralement faible [2]. La tomodensitométrie abdominale est un examen performant pour le diagnostic. Elle permet de diagnostiquer une complication associée comme une ischémie ou une perforation [6]. La coloscopie peut être réalisée montrant le volvulus et une ischémie pariétale colique plus ou moins profonde [8,9]. Une détorsion endoscopique est faisable en absence d'ischémie sévère mais comporte un risque non négligeable de perforation [10]. Le traitement a trois buts, il s'agit de lever l'obstacle par une détorsion, si cela est possible, de traiter les complications évolutives et de prévenir la récidive [11]. Il demeure un sujet de controverse. L'hémicolectomie droite avec anastomose pri-maire est recommandée par plusieurs équipes même en absence de nécrose colique car elle supprime le risque de récidive [12-15]. La cæcostomie est efficace pour la prévention des récidives mais comporte un risque élevé d'infection de paroi et expose au risque de fistule digestive nécessitant une intervention de fermeture. Les complications infectieuses sont moins fréquentes avec les cæcopexies mais les récidives sont plus fréquentes [16]. La voie d'abord cœlioscopique [17] est rarement utilisée en urgence du fait de la distension du cæcum et des difficultés d'exposition. Elle pourrait être réalisée après une détorsion et une exsufflation endoscopique.

## Conclusion

La variabilité de la topographie de la région iléo-cæcale et de ses accolements péritonéaux est importante. Le volvulus du cæcum doit être évoqué chez les malades qui ont une douleur abdominale aiguë notamment lorsqu'il y a des signes radiologiques évocateurs. Une prise en charge rapide et adaptée est nécessaire pour diminuer le risque de morbidité et mortalité.

## Conflits d’intérêts

Les auteurs ne déclarent aucun conflits d’'intérêts.
